# Supplementary material for: Patterns of Intron Gain and Loss in Fungi
Source: PLoS Biol. 2004 Nov 30;2(12):e422. doi: 10.1371/journal.pbio.0020422 (PMC532390; doi:10.1371/journal.pbio.0020422)
Supplement: Table S1 — Also available at http://genes.mit.edu/NielsenEtAl/. (4.3 MB ZIP). [file pbio.0020422.st001.zip › NielsenEtAl/html/566.html]

AN6499.1.NCU06211.1.MG08835.1.FG02504.1


```
 CLUSTAL W (1.82) Multiple Sequence Alignments - Introns Inserted


Sequence 1: NCU06211.1	330 aa
Sequence 2: MG08835.1	330 aa
Sequence 3: FG02504.1	330 aa
Sequence 4: AN6499.1	320 aa
Alignment Length: 334 aa
Number Identitical Residues: 211 aa
Alignment Score (without introns) 9401


MG08835.1 	MVKAV1VAGAAGGIGQ0PLSLLLKLCPLVDELALYDVVNTPGVAADLSHISSNA0KIAGY
NCU06211.1	MVKAV1VAGAAGGIGQ0PLSLLLKLSPLVDELALYDVVNTPGVAADLSHISSPA0KTTGY
FG02504.1 	MVKAV1VAGASGGIGQ0PLSLLLKTSPHIDELALYDVVNTPGVATDLSHISSRA0KTTGY
AN6499.1  	-----~---------M0PLSLLLKASPFIDELALYDVVNTPGVAADLSHISSVA0KISGY
          	                 ******* .* :***************:******* * * :**

MG08835.1 	LPKDDGGKKALKDADLIVIPAGVPR1KPGMTRDDLFNINAGIVKGLIEIAAEVAPKAFIL
NCU06211.1	LPPNDGAKAAFKDADIIVIPAGIPR1KPGMTRDDLFNINAGIVKGLIEVAADVAPNAFIL
FG02504.1 	LPANDGAKAAFKDADIIVIPAGIPR1KPGMTRDDLFNINAGIVKGLIEVAAEVAPKAFIL
AN6499.1  	LPKEDGLKNALTGTDIVVIPAGIPR1KPGMTRDDLFKINAGIVRDLVKGIAEYSPKAFIL
          	** :** * *:..:*::*****:** **********:******:.*::  *: :*:****

MG08835.1 	VISNPVNSTVPISAEVLKAKGVFNPQRLFGVTTLDIVRAETFVAEIAGKSNPQELTVPVI
NCU06211.1	VISNPVNSTVPISAEVLKAKGVFNAQRLFGVTTLDIVRAETFVAEIAGNSNPQELVVPVI
FG02504.1 	VISNPVNSTVPISAEVLKAKGVFNPQRLFGVTTLDIVRAETFVAEITGKANPQELTIPVI
AN6499.1  	IISNPVNSTVPIAAEILKAAGVFDPARLFGVTTLDVVRAETFTQEFSGQKDPSAVTVPVV
          	:***********:**:*** ***:. *********:******. *::*: :*. :.:**:

MG08835.1 	GGHSGETIVPLFSQVKPAVTIPDDKYDALVNR~VQFGGDEVVKAKDGAGSATLSMAYAGY
NCU06211.1	GGHSGETIVPLFSKVSPSVTIPDDKYDALVNR~VQFGGDEVVKAKEGLGSATLSMAYAGY
FG02504.1 	GGHSGETIVPLFSKASPSVQIPDDKYDALVNR~IQFGGDEVVKAKDGAGSATLSMAYAGF
AN6499.1  	GGHSGETIVPLFSKVSPAFQIPADKYDALVNR1VQFGGDEVVKAKDGAGSATLSMAFAGF
          	*************:..*:. ** ********* :***********:* ********:**:

MG08835.1 	R2----~FAEKLLKAIKGAKGLVEPSYVYLPGVPGGEAIAKKTGCDFFSVPIELG0PNGA
NCU06211.1	R~----~FAEKLLKAAKGAKGLVEPTYVYLPGIPGGKEIAEKTGVDFFSVPVELG0PNGA
FG02504.1 	R2----~FAEKVLRAVKGEKGLVEPSYVYLPGVPGGEAIAKETGCDFFSVPIELG0PNGA
AN6499.1  	R~SGIL2FAESVIKASKGQSGIVEPSYVYLPGVPGGADIAKATGVNFFSTPVELG0PNGV
          	* :.   ***.:::* ** .*:***:******:***  **: ** :***.*:*** ***.

MG08835.1 	EKAHDVLGELTSKEQTLLEAAVNGLKGNIQKGVQFVNSPPQK
NCU06211.1	EKAIDILGDITEKEKTLLEAAIKGLKGNIQKGVDFAHNPPQK
FG02504.1 	EKATNPFEGITEKEKALLAKATEGLKGNITKGVSFVHNPPQK
AN6499.1  	QKAINILDGITDAEKKLLDTAIKGLKGNIDKGVEFAQSPPPK
          	:** : :  :*. *: **  * :****** ***.*.:.** *
```
